# Identification of 13 Key Genes Correlated With Progression and Prognosis in Hepatocellular Carcinoma by Weighted Gene Co-expression Network Analysis

**DOI:** 10.3389/fgene.2020.00153

**Published:** 2020-02-28

**Authors:** Yang Gu, Jun Li, Deliang Guo, Baiyang Chen, Pengpeng Liu, Yusha Xiao, Kang Yang, Zhisu Liu, Quanyan Liu

**Affiliations:** Department of Hepatobiliary and Pancreas Surgery, Zhongnan Hospital of Wuhan University, Wuhan, China

**Keywords:** quantitative real-time PCR, bioinformatics, weighted gene co-expression network analyses, hepatocellular carcinoma, biomarker

## Abstract

Hepatocellular carcinoma (HCC) remains hard to diagnose early and cure due to a lack of accurate biomarkers and effective treatments. Hence, it is necessary to explore the tumorigenesis and tumor progression of HCC to discover new biomarkers for clinical treatment. We performed weighted gene co-expression network analysis (WGCNA) to explore hub genes that have high correlation with clinical information. In this study, we found 13 hub genes (*GTSE1*, *PLK1*, *NCAPH*, *SKA3*, *LMNB2*, *SPC25*, *HJURP*, *DEPDC1B*, *CDCA4*, *UBE2C*, *LMNB1*, *PRR11*, and *SNRPD2*) that have high correlation with histologic grade in HCC by analyzing TCGA LIHC dataset. All of these 13 hub genes could be used to effectively distinguish high histologic grade from low histologic grade of HCC through analysis of the ROC curve. The overall survival and disease-free survival information showed that high expression of these 13 hub genes led to poor prognosis. Meanwhile, these 13 hub genes had significantly different expression in HCC tumor and non-tumor tissues. We downloaded GSE6764, which contains corresponding clinical information, to validate the expression of these 13 hub genes. At the same time, we performed quantitative real-time PCR to validate the differences in the expression tendencies of these 13 hub genes between HCC tumor tissues and non-tumor tissues and high histologic grade and low histologic grade. We also explored mutation and methylation information of these 13 hub genes for further study. In summary, 13 hub genes correlated with the progression and prognosis of HCC were discovered by WGCNA in our study, and these hub genes may contribute to the tumorigenesis and tumor progression of HCC.

## Introduction

Hepatocellular carcinoma is one of the most malignant tumors and is the third leading cause of cancer death in the world (Altekruse et al., 2009; GBD 2015 Mortality and Causes of Death Collaborators, 2016; Li et al., 2017). HCC is extremely hard to diagnose at an early stage, and there are currently no effective treatments for patients with advanced-stage HCC. Surgery remains the most common treatment for HCC; nevertheless, the decade survival rate is not increasing obviously (Waghray et al., 2015; Wallace et al., 2015). HBV infection is one of the most common factors in HCC in the world (de Martel et al., 2015). Patients with higher TNM stage and histologic grade have a worse prognosis compared with those for whom they are lower (Ikeda et al., 1993). It is crucial to reach an understanding of the pathological changes from normal liver to HCC. Meanwhile, effective early detection and diagnostic methods are urgently needed for improving the prognosis and treatment of HCC patients.

The use of microarray as a high-throughput platform for gene expression analysis has been soaring in recent years (Murakami et al., 2006; Li et al., 2018). Large numbers of changed genes can be detected easily by this technology, and these can be analyzed to identify critical significant genes as biomarkers (Giulietti et al., 2018; Tang et al., 2018). There are plenty of public free databases, such as TCGA and the GEO, from which abundant information can be excavated via various bioinformatics methods. WGCNA, a method of building a multiple gene co-expression model, can be used to cluster similarly expressed genes and analyze the relationships of differential character and phenotype within modules (Langfelder and Horvath, 2008; Giulietti et al., 2016; Wang Y. et al., 2019). WGCNA can divide genes into a model or network based on pairwise correlations between genes based on their similar expression profiles, and these models can correlate to different information on HCC. Compared to conventional methods, WGCNA establishes a more meaningful correlation between gene expression and clinical information. In this study, we identified 13 hub genes that have strong relationships with histologic grade in HCC by WGCNA, using the TCGA LIHC dataset. The ROC curve showed that almost all hub genes could effectively identify high histologic grade and low histologic grade. Meanwhile, the overall survival and disease-free survival of almost hub genes have significance in 10 years. Almost all of these 13 hub genes had higher expression in HCC tumor tissues compared with non-tumor tissues. After downloading GSE6764, we validated that these hub genes had the same expression tendencies as shown by the results of TCGA. Quantitative real-time PCR was also used to validate the differential expression of these hub genes between HCC tumor tissues and non-tumor tissues and high histologic grade and low histologic grade.

## Materials and Methods

### Study Approval

None of the patients had been subjected to any neoadjuvant therapy before surgery. Informed consent was obtained from each patient. The protocols used in this study were approved by the Protection of Human Subjects Committee of Zhongnan Hospital. All research was performed in accordance with relevant guidelines and regulations.

### Data Collection and Processing

**FIGURE 1:**
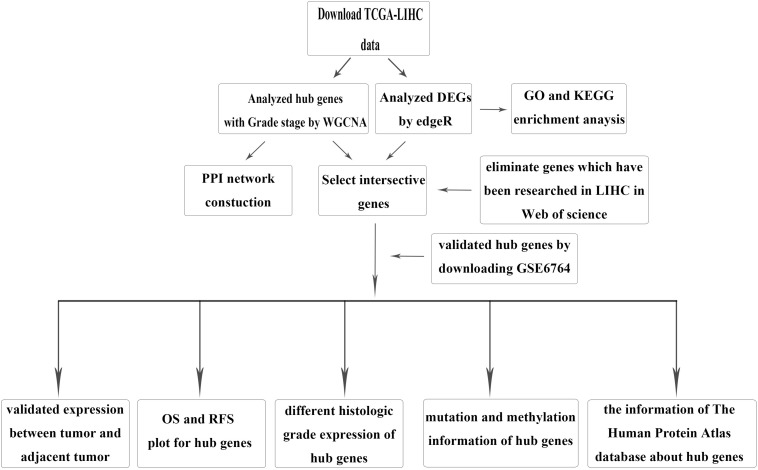
Flow chartpc of data download, processing, analysis, and validation.

The brief study design is shown in Figure 1. The RNA-sequencing (RNA-seq) data and corresponding clinical information for HCC were downloaded from TCGA LIHC dataset, which included 371 tumor samples and 50 adjacent tumor samples. The clinical information was comprised of event, TNM stage, histologic grade, new event, diagnosis age, and HBV infection. Due to some clinical information being missing, we used not available (NA) to replace the missing values for WGCNA. After normalization and filtering with the “edgeR” package (Wilson and Miller, 2005; Robinson et al., 2010), *p*-value < 0.05 and | logFC (FC)| > 1 were used for screening DEGs. The validation dataset GSE6764, which contained 10 normal liver tissues, 8 very early HCC tissues, 10 early HCC tissues, 7 advanced HCC tissues, and 10 very advanced HCC tissues, was downloaded from the GEO.

### GO Enrichment and KEGG Pathway Analysis

The GO database, which is comprised of MF, BP, and CC, is used for observing gene annotation (Harris et al., 2004). The KEGG is a database resource for understanding high-level functions and utilities of the biological system (Kanehisa et al., 2016). DAVID^1^ is an online website that can provide a comprehensive set of functional annotation tools through which investigators can understand the biological meaning behind a large list of genes (Huang da et al., 2009). In this study, DAVID 6.7 was used to analyze the GO enrichment and KEGG pathways of all DEGs. The *p*-value < 0.05 was considered statistically significant.

### Weighted Gene Co-expression Network Construction

We selected the top 25% variance genes, which included 4938 genes, to construct a co-expression network with the “WGCNA” package in R. Elimination of outliers is the first step in this analysis. Next, we select an appropriate soft-thresholding power that would ensure scale-free topology, *R*^2^ = 0.9. The adjacency was then transformed into a TOM by using TOM similarity and the corresponding dissimilarity (dissTOM). Finally, at least 30 co-expressed genes were aggregated into different MEs by the dynamic tree cut method. We decided on a cut line (<0.25) for merging highly similar modules to make the modules more compact. A cluster dendrogram was used to show the result.

### Identification of Clinical Significant Modules

The correlations between clinical information and MEs were calculated by WGCNA in R. We selected the most significant and correlated modules with HCC. In this study, the genes in blue and turquoise MEs with the histologic grade of HCC were our next research objects. Meanwhile, two concepts of GS and MM were proposed in our study. Genes that have high significance for clinical information and MM were confirmed by these two parameters.

### Hub Gene Identification and Verification

The connectivity of genes was measured by the absolute value of the Pearson’s correlation. Genes with high within-module connectivity were considered to be hub genes of the modules (cor.geneModuleMembership > 0.85). Hub genes in given modules tended to have a strong correlation with certain clinical information, which was measured by the absolute value of the Pearson’s correlation (cor.geneTraitSignificance > 0.2). Next, we intersected hub genes in MEs with all DEGs via the edgeR package. At the same time, we eliminated some hub genes that have been researched multiple times in HCC. We definitely considered these hub genes significant by this filter. To validate the functions of these hub genes, the GSE6764 microarray was downloaded from the GEO database in NCBI.

### Survival Analysis and ROC Analysis of Hub Genes

To identify whether these hub genes have deeper value, we searched the GEPIA website^2^ for overall survival and disease-free survival analysis of all of the hub genes (Tang et al., 2017). The log-rank test was used to show statistical significance. Meanwhile, the AUC, calculated by *R*, was used to differentiate different histologic grades (high histologic and low histologic) of HCC.

### Mutation and Methylation Information of Hub Genes

We searched the cBioPortal website^3^ to identify whether these hub genes have epigenetics information about mutation and methylation (Cerami et al., 2012). The corresponding figures were downloaded from that website.

### Immunohistochemical Information of Hub Genes

To explore the expression of these hub genes in HCC and normal tissues, we searched the Human Protein Atlas public database^4^ for IHC information.

### Tissue Collection and Quantitative Real-Time PCR

A total of 16 pairs of HCC and corresponding adjacent tissues were obtained from 16 HCC patients after surgery in Zhongnan Hospital, Wuhan University. The histologic grades of all HCC tissues were identified by the pathology department in our hospital. All samples were stored with RNA protective reagent at −80°C. Total RNA was extracted from HCC tissues by TRIzol reagent (Invitrogen, Carlsbad, CA, United States). Extracted total RNA was quantified using a NanoDrop spectrophotometer (Thermo Scientific Inc.) at 260 and 280 nm. RNA was used for reverse transcription when *A*_260_/*A*_280_ = 2.0. The reverse transcription was processed with the PrimeScript RT reagent Kit with gDNAEraser (Takara, Tokyo, Japan). Quantitative real-time PCR was performed with the SYBR Green PCR kit (Toyobo, Osaka, Japan) using the CFX Connect Real-Time PCR Detection System (Bio-Rad, United States) after setting up the appropriate protocol. The GAPDH was used as an internal control. The 2^–ΔΔ^ ^*Ct*^ method was used to analyze the results of quantitative real-time PCR. All the primers were designed by the website PrimerBank^5^ and are listed in Supplementary Table 1.

## Results

### Identification of Differentially Expressed Genes

**FIGURE 2:**
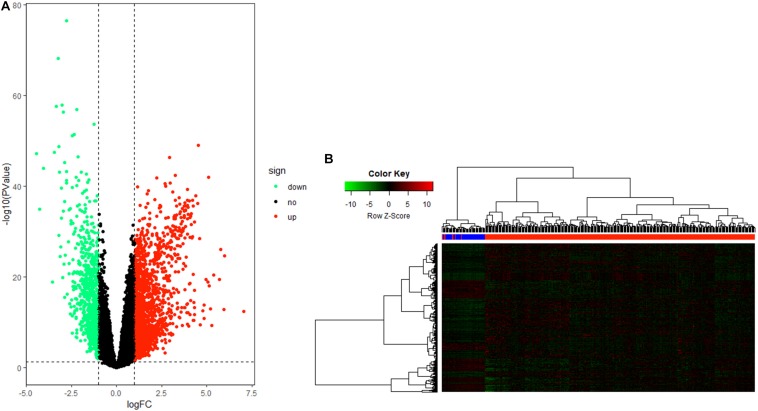
Differentially expressed gene (DEG) identification. **(A)** Volcano plot of all genes in hepatocellular carcinoma (HCC). **(B)** Heatmap plot of all DEGs.

After data processing, a total number of 2356 significant DEGs, including 789 down-regulated and 1567 up-regulated genes, were identified between HCC tissue and adjacent tumor tissue by the “edgeR” package in R. Volcano and heatmap plots were used to show this result (Figures 2A,B).

### GO and KEGG Pathway Enrichment Analysis of DEGs

**FIGURE 3:**
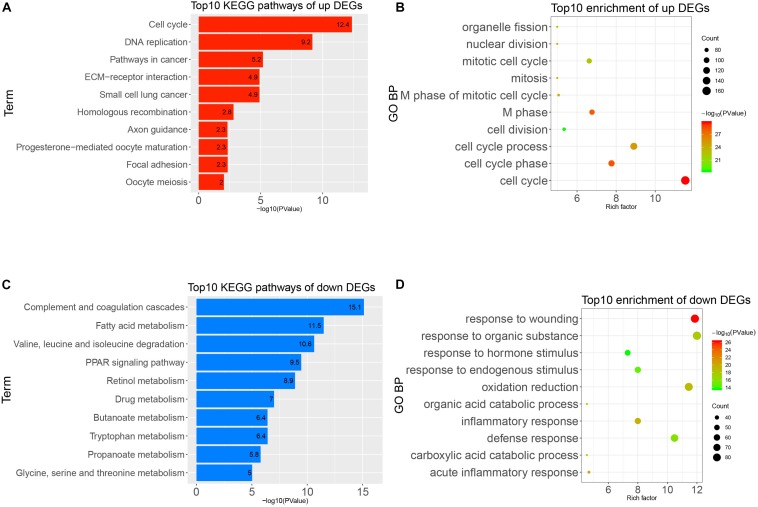
Gene Ontology (GO) and Kyoto Encyclopedia of Genes and Genomes (KEGG) pathways analysis of all differentially expressed genes (DEGs). **(A)** Top 10 enrichments of up-regulated DEGs by GO biological process. **(B)** Top 10 KEGG pathways of up-regulated DEGs. **(C)** Top 10 enrichment of down-regulated DEGs by GO biological process. **(D)** Top 10 KEGG pathways of down-regulated DEGs.

To obtain a deeper understanding of the annotation and function of all of the DEGs, we put all of these DEGs into DAVID to analyze significant GO and KEGG pathways. The up-regulated DEGs were remarkably enriched in cell cycle, M phase, M phase of mitotic cell cycle, mitotic cell cycle, and other BP (Figure 3A). The KEGG enrichment analysis identified cell cycle, DNA replication, pathways in cancer, and other pathways (Figure 3B). Meanwhile, the down-regulated DEGs were mainly enriched in response to wounding, acute inflammatory response, oxidation–reduction, and other BP (Figure 3C). The KEGG enrichment results of down-regulated DEGs are complement and coagulation cascades, fatty acid metabolism, PPAR signaling pathway, and other pathways (Figure 3D). It seems that the disorder of these pathways possibly reflected the intricate pathological mechanism of HCC.

### Weight Gene Co-expression Network Construction and Key Module Identification

**FIGURE 4:**
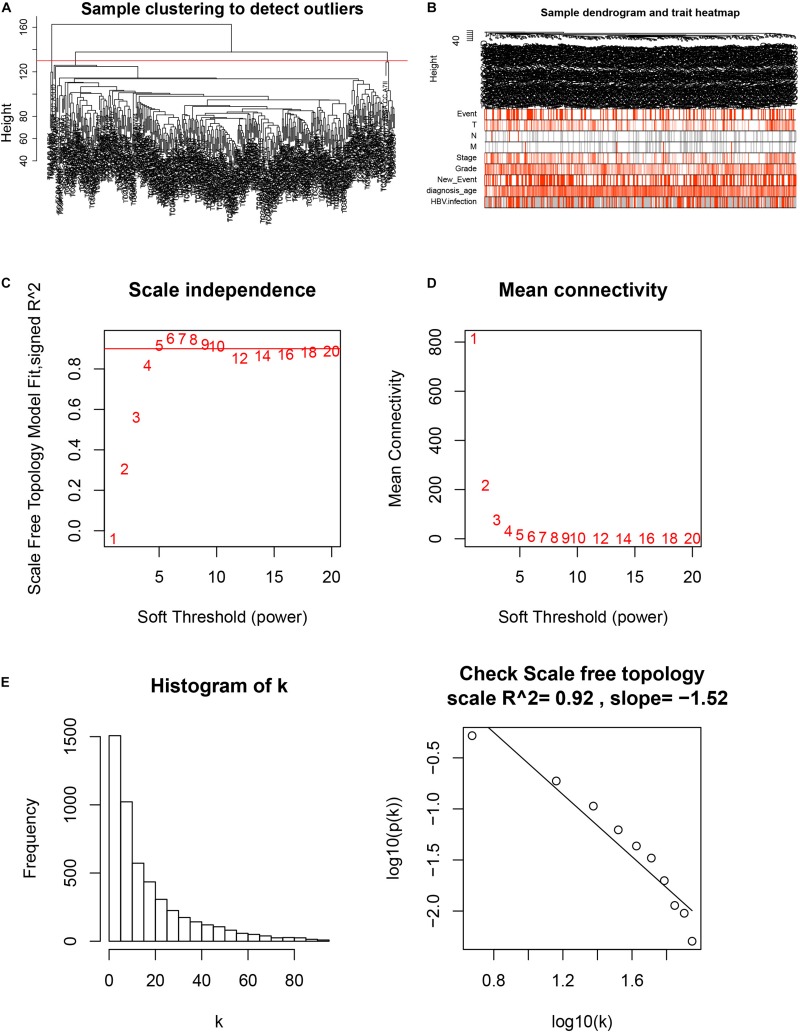
Sample clustering and determination of thresholding power in weighted gene co-expression network analysis (WGCNA). **(A)** Sample clustering to detect outliers. **(B)** Sample dendrogram and trait heatmap. **(C)** Analysis of the scale-free fit index for various soft-thresholding powers (β). **(D)** Analysis of the mean connectivity for various soft-thresholding powers. **(E)** Checking the scale-free topology when β = 5.

**FIGURE 5:**
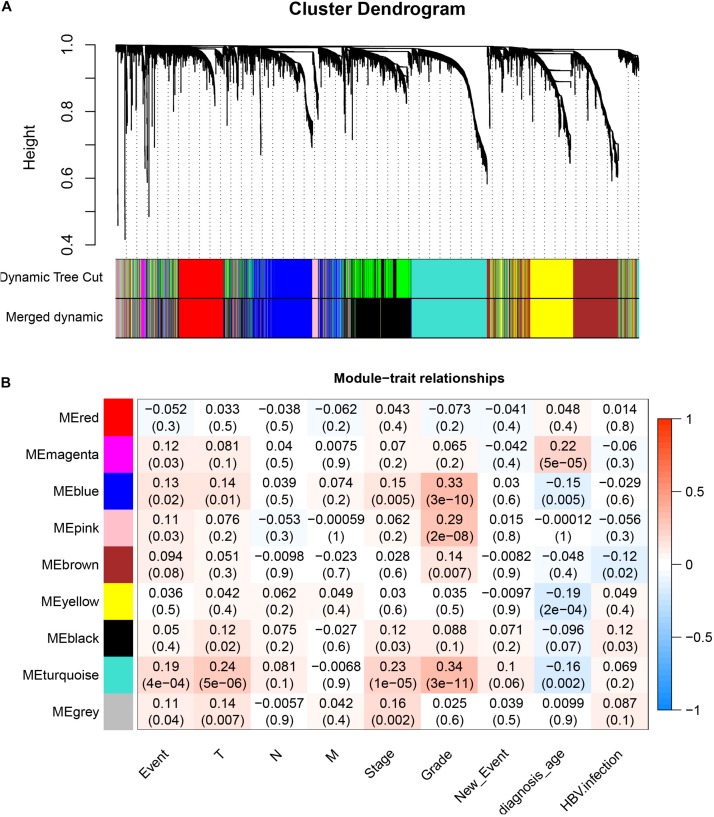
Identification of modules associated with histologic grade of HCC. **(A)** Dendrogram of all differentially expressed genes clustered based on a dissimilarity measure (1–TOM). **(B)** Heatmap of correlation between module eigengenes and clinical information for HCC.

**FIGURE 6:**
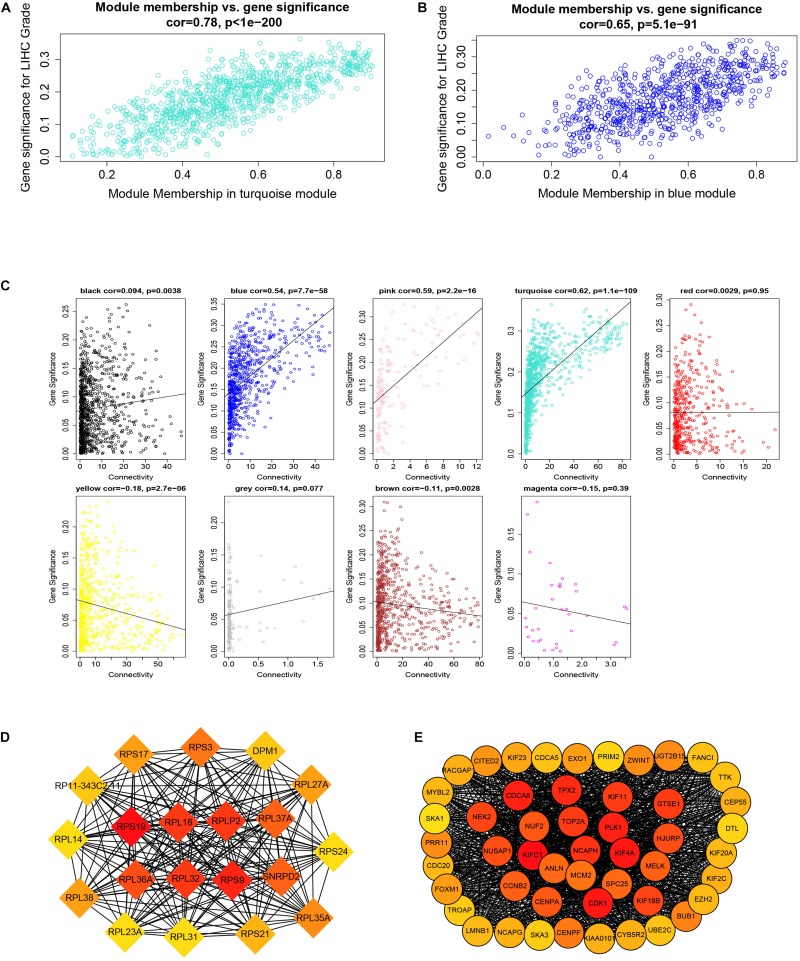
Module membership and corresponding gene significance and protein–protein interaction (PPI) network construction of two key modules. **(A)** Module membership in the turquoise module. **(B)** Module membership in the blue module. **(C)** Relationship between the connectivity of all modules and gene significance. **(D)** Top 20 hub genes in the blue module according to cytoHubba analysis. **(E)** Top 50 hub genes in the turquoise module according to cytoHubba analysis.

After downloading the FPKM value expression matrix of all HCC samples, we selected the top 25% variance genes, including 4938 for WGCNA. To eliminate outliers, we chose 130 for the cut tree height for the samples (Figure 4A). The number of HCC samples under the red line was 352 after clustering. The sample dendrogram and trait heatmap of 352 samples in our study are shown in Figure 4B. We chose the power of β = 5 (scale-free *R*^2^ = 0.92, slope = −1.52) as the soft-thresholding to construct a scale-free network (Figures 4C,D). By gathering similarly expressed genes, a total of nine modules were identified, namely black module [947], blue module [748], brown module [735], gray module [160], magenta module [35], pink module [160], red module [460], turquoise module [1023], and yellow module [670] (Figure 5A). The MEs in the blue and turquoise modules showed a higher correlation with histologic grade of HCC (*R*^2^ = 0.33, *p* = 3*e*−10; *R*^2^ = 0.34, *p* = 3*e*−11) (Figure 5B). We therefore chose these two modules for further research. The module membership and gene significance for LIHC stage in turquoise and blue module were showed in Figures 6A,B. The relationship between connectivity in modules and gene significance was showed in Figure 6C.

### Identification of Hub Genes

**FIGURE 7:**
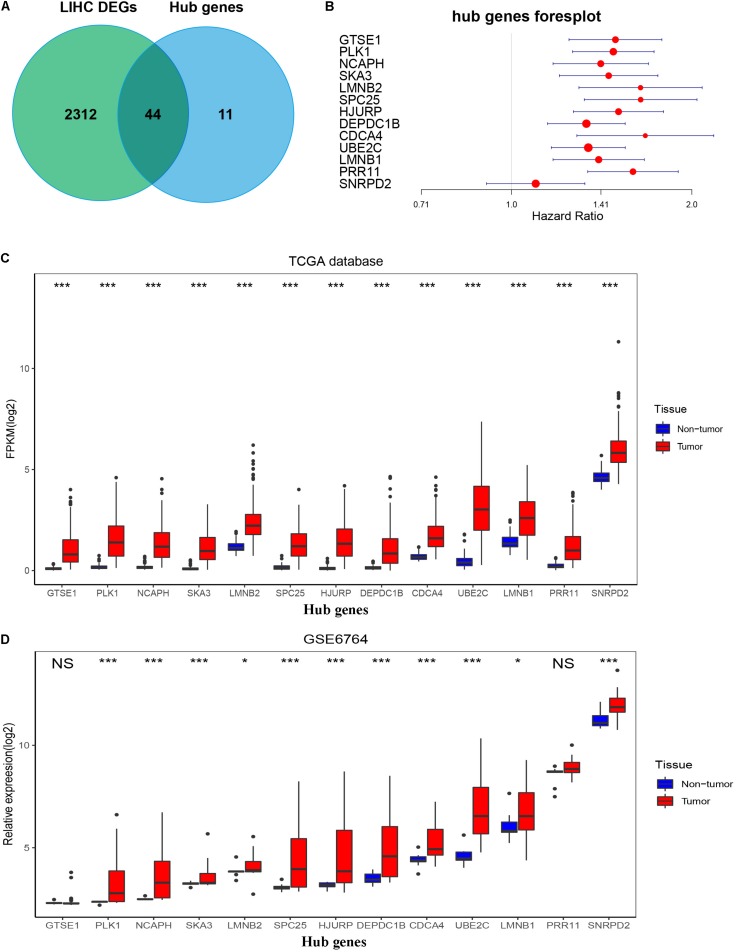
Confirmation and validation of 13 hub genes differentially expressed in tumor tissues and non-tumor tissues in the TCGA database and GSE6764. **(A)** The 44 common genes between all DEGs according to the edgeR package and hub genes according to WGCNA. **(B)** Forest plot of 13 hub genes. **(C)** The boxplot of 13 hub genes differentially expressed in tumor and non-tumor tissues of HCC in the TCGA database. *P*-value was calculated by *t*-test. *p < 0.05, ***p < 0.001. **(D)** Boxplot of 13 hub genes differentially expressed in tumor and non-tumor tissues of HCC in GSE6764. *P*-value was calculated by *t*-test. *p < 0.05, ***p < 0.001; NS, no significance.

By setting up cor.geneModuleMembership > 0.85 and cor.geneTraitSignificance > 0.2, there are 9 hub genes in the blue module and 46 hub genes in the turquoise module (Supplementary Table 2). The PPI networks of all genes in the two modules were, respectively, constructed by Cytoscape. We used the cytoHubba app to calculate the degree of each node. The nine hub genes were included in the top 20 hub genes in the blue module according to cytoHubba (Figure 6D). The 46 hub genes were also included in the top 50 hub genes in the turquoise module according to cytoHubba (Figure 6E). Next, we took the 44 intersecting genes from the two module hub genes and all DEGs in HCC (Figure 7A). Considering that some of the hub genes have already been reported on and researched in HCC, such as *NCAPG*, *TTK*, *TOP2A*, *CDC20*, *CDK1*, and so on (Wong et al., 2009; Li et al., 2014; Liu et al., 2015; Wu et al., 2018; Zhang et al., 2018), we finally chose 13 hub genes (*GTSE1*, *PLK1*, *NCAPH*, *SKA3*, *LMNB2*, *SPC25*, *HJURP*, *DEPDC1B*, *CDCA4*, *UBE2C*, *LMNB1*, *PRR11*, and *SNRPD2*) on which little research had been done regarding HCC to continue our deeper exploration.

### Validation of Hub Genes

**FIGURE 8:**
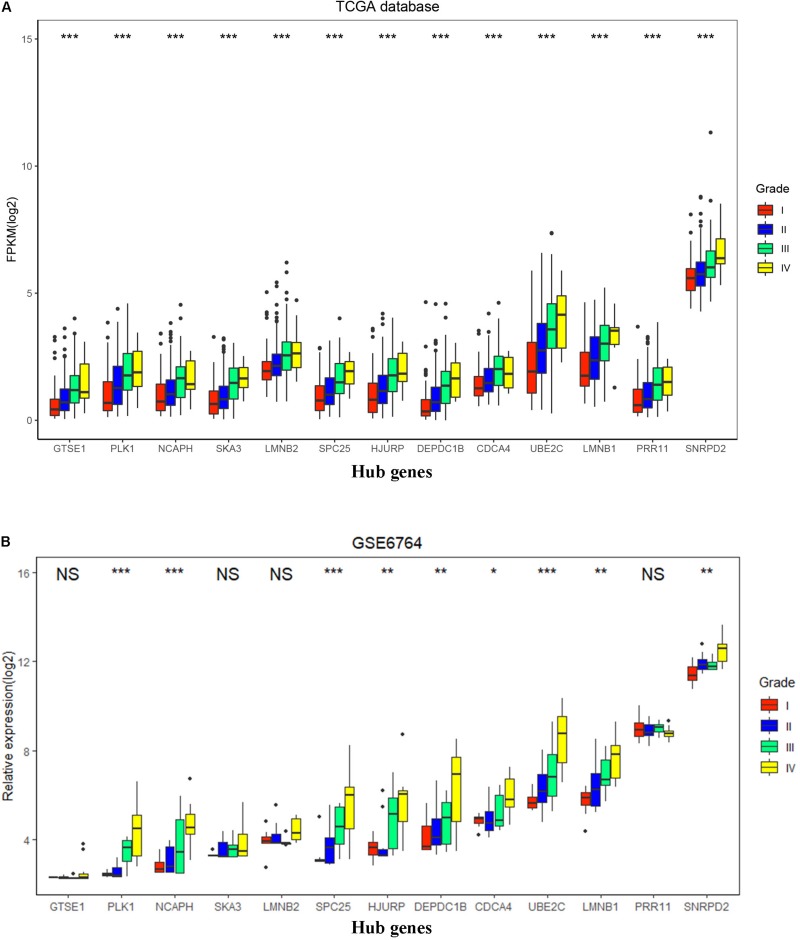
Hub gene expression in different histologic grades in The Cancer Genome Atlas (TCGA) database and GSE6764. **(A)** Boxplot of 13 hub genes in histologic grades I–IV in the TCGA database. *P*-value was calculated by one-way ANOVA. ***p < 0.001. **(B)** Boxplot of 13 hub genes in histologic grades I–IV in GSE6764. *P*-value was calculated by one-way ANOVA. *p < 0.05, **p < 0.01, ***p < 0.001; NS, no significance.

To validate the expression of these 13 hub genes in tumor tissue and adjacent tissue, we downloaded GSE6764, in which there are 11 hub genes that have the same tendency and statistical significance compared with the TCGA database (Figures 7C,D). All of these hub genes belonged to the set of adverse factors in HCC (Figure 7B). To validate different histologic grade expression, four groups (very early HCC, early HCC, advanced HCC, and very advanced HCC) in GSE6764 were considered to approximate histologic grades I–IV, and most of the hub genes had significance in a one-way ANOVA test (Figures 8A,B).

### Survival and ROC Analysis

**FIGURE 9:**
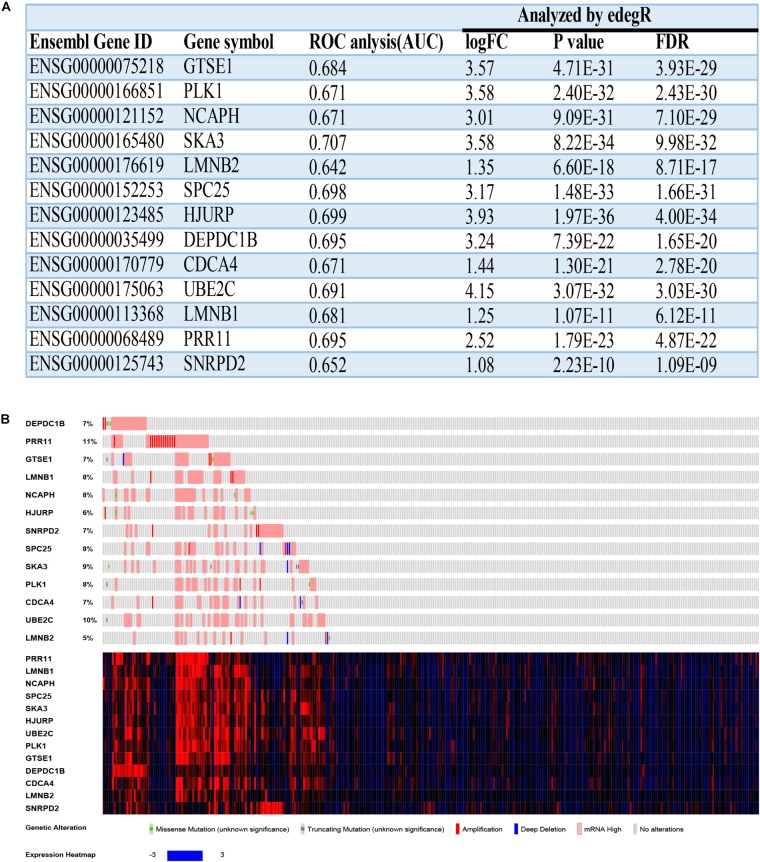
The basal information and mutation information for 13 hub genes. **(A)** Information on 13 hub genes. ROC, receiver operating characteristic; AUC, area under curve; FC, fold change; FDR, false discovery rate. **(B)** Mutation information on 13 hub genes in the cBioPortal website.

The overall survival and disease-free survival of these 13 hub genes were analyzed by using the GEIPA online database. Nearly all of them had a poor prognosis when highly expressed on the basis of log-rank test analysis (Supplementary Figures 1–5). The ROC curve was used to measure early HCC (Grades I and II) and advanced HCC (Grades III and IV). The AUC of almost all hub genes exceed 0.65, which meant that these hub genes could effectively differentiate early HCC and advanced HCC (Supplementary Figures 6–8. The AUCs of hub genes are listed in Figure 9A).

### Mutation and Methylation Information

To find deeper information on these hub genes, we searched the cBioPortal website to identify whether these hub genes have epigenetics information about mutation and methylation available. Unfortunately, all hub genes had no obvious mutation events (Figure 9B). Meanwhile, *PRR11* had more amplifications compared with the other hub genes, which could explain its high expression in HCC. The mRNA expressions (*Z*-scores) of more than half of the 13 hub genes had negative correlations (Pearson correlation > | −0.3|, *p* < 0.05) with methylation (HM450) (Supplementary Figures 9, 10). Among these results, the correlation of *DEPDC1B* even reached −0.52, which revealed that methylation of the promoter region of *DEPDC1B* probably regulated expression of the corresponding mRNA.

### Immunohistochemical Information

To obtain precise results, we chose the same antibody for each hub gene between HCC and normal tissue. The results of the IHC analysis and figures are shown in Supplementary Figures 11, 12.

### Tissue Validation by Quantitative Real-Time PCR

The results of quantitative real-time PCR showed that 12 hub genes had significantly different expressions in HCC tissues and adjacent tissues on the basis of paired *t*-test. However, *PRR11* showed no significant differential expression between HCC tissues and adjacent tissues (Supplementary Figures 13, 14). Meanwhile, the expression of 13 hub genes in high histologic grade was higher than that in low histologic grade, except *SKA3*, which demonstrated that the results of WGCNA made sense (Supplementary Figure 14).

## Discussion

Hepatocellular carcinoma seriously impacts human health, and it is likely to recur after surgery combined with other therapy (Worns and Galle, 2010). The 10-year survival rate of patients will observably decrease when metastasis is found, even if optimal treatments are used. It is crucial to explore the etiological and biological mechanisms involved in the occurrence and development of HCC. Better biomarkers for predictive and prognostic molecules for HCC are urgently required. The RNA-seq-expression and clinical information of HCC from the TCGA database are correlated by WGCNA.

In this study, we constructed protein–protein networks to show the top 20 and 50 genes in the blue and turquoise modules by calculating degree in Cytoscape. Through identification of DEGs, WGCNA, and filtering out some of the genes, we finally screened out 13 hub genes on which few studies had been conducted in HCC. These hub genes were not only expressed differently in HCC and adjacent tissue but also had significance with overall survival and disease-free survival. Meanwhile, almost all of the hub genes could effectively distinguish early grade HCC from advanced HCC, which implied that these hub genes play an important role in the progress of HCC. Genome-wide DNA methylation is well known, but its emergence and development mechanisms remain mysterious (Meissner et al., 2008; Lister et al., 2009). By searching cBioPortal, we found that the correlation between *DEPDC1B* expression and corresponding methylation reached −0.52 in HCC. *DEPDC1B*, as a protein that accumulates in G2, coordinates de-adhesion events and cell-cycle progression at mitosis (Marchesi et al., 2014). A previous study showed that *DEPDC1B* was a guanine nucleotide exchange factor and induced both cell invasion and migration in oral cell lines (Su et al., 2014). However, the function of *DEPDC1B* in HCC has not been reported yet.

When the expressions of the subset of the hub genes were validated in GSE6764, we found that *GTSE1* and *PRR11* showed no obvious differences between HCC and normal tissues. At the same time, *GTSE1*, *SKA3*, *LMNB2*, and *PRR11* had no obvious tendency between different histologic grades. *GTSE1* and *PRR11* have been reported in various types of tumor. High expression of *GTSE1* can promote breast cancer growth and metastasis. Moreover, *GTSE1* can cause multidrug resistance in breast cancer cells (Lin et al., 2019). In Tongue Squamous Cell Carcinoma (TSCC) cells, *PRR11* can promote proliferation and invasion by regulating p21, p27, CDK2, and Cyclin A to facilitate S phase progression (Wang C. et al., 2019). Recently, a study showed that down-regulation of *SKA3* could inhibit HCC proliferation and invasion *in vivo* and *in vitro*. On the basis of gene enrichment analysis (GSEA), *SKA3* was enriched in the cell cycle and P53 signaling pathways and these results were verified (Hou et al., 2019). Moreover, it has been reported that *SKA3* can facilitate PI3K/AKT signaling pathway to promote cell cycle and progression in cervical cancer (Hu et al., 2018).

Compared with the HCC samples in the TCGA database, GSE6764 had few samples in each group, which may lead to this incomplete result. Besides, we could not find paired patient samples of HCC and normal tissue for immunohistochemistry due to the limitation of the Human Protein Atlas database. By quantitative real-time PCR in 16 paired tissues, we found that all hub genes had significance between tumor tissues and non-tumor tissues except *PRR11*. Meanwhile, all hub genes except *SKA3* had significance for distinguishing high histologic grade from low histologic grade in HCC.

It is necessary for us to enlarge our sample set to validate the expression levels of these hub genes. However, we could not proceed further with the research because of various limitations. We hope that other researchers will be able to uncover the specific functions and mechanisms of these 13 hub genes in HCC, especially *DEPDC1B*.

## Conclusion

In conclusion, this study aimed to identify hub genes involved in HCC by WGCNA of data from the TCGA database and concluded that 13 functional genes (*GTSE1*, *PLK1*, *NCAPH*, *SKA3*, *LMNB2*, *SPC25*, *HJURP*, *DEPDC1B*, *CDCA4*, *UBE2C*, *LMNB1*, *PRR11*, and *SNRPD2*) are highly differentially expressed in tumor and non-tumor tissues. Further bioinformatic analysis showed that these hub genes can act as biomarkers of HCC progression and prognosis. At the same time, experimental assay was performed to validate these hub genes. However, further molecular experiments on these hub genes in HCC, *in vivo* and *in vitro*, need to be carried out.

## Data Availability Statement

The RNA-seq data and corresponding clinical information of HCC were downloaded from TCGA. The validation dataset accession GES6764 microarray was downloaded from GEO database in NCBI. All remaining raw data supporting the conclusions of this manuscript will be made available by the authors, without undue reservation, to any qualified researcher.

## Ethics Statement

The studies involving human participants were reviewed and approved by the Protection of Human Subjects Committee of Zhongnan Hospital. The patients/participants provided their written informed consent to participate in this study.

## Author Contributions

YG, QL, and ZL designed this study. YG, JL, DG, and KY analyzed the data and wrote the manuscript. YG, BC, PL, and YX conducted the quantitative real-time PCR. All authors reviewed the manuscript.

## Conflict of Interest

The authors declare that the research was conducted in the absence of any commercial or financial relationships that could be construed as a potential conflict of interest.

## Footnotes

1 https://david-d.ncifcrf.gov/

2 http://gepia.cancer-pku.cn/

3 http://www.cbioportal.org/

4 https://www.proteinatlas.org/

5 https://pga.mgh.harvard.edu/primerbank/

## Abbreviations

AUC: area under curve
BP: biological process
CC: cellular component
DEGs: differentially expressed genes
dissTOM: dissimilarity topological overlap matrix
FC: fold change
FPKM: fragments per kilobase of transcript per million fragments mapped
GEIPA: Gene Expression Profiling Interactive Analysis
GEO: Gene Expression Omnibus
GO: Gene Ontology
GS: gene significance
HBV: Hepatitis B virus
HCC: hepatocellular carcinoma
IHC: immunohistochemical
KEGG: Kyoto Encyclopedia of Genes and Genomes
MEs: module eigengenes
MF: molecular function
MM: module membership
NCBI: National Center for Biotechnology Information
PCR: polymerase chain reaction
PPI: protein–protein interaction
ROC: receiver operating characteristic
TCGA: The Cancer Genome Atlas
TOM: topological overlap matrix
WGCNA: Weighted Gene Co-Expression Network Analysis.

## References

[B1] Altekruse S. F. McGlynn K. A. Reichman M. E. (2009). Hepatocellular carcinoma incidence, mortality, and survival trends in the United States from 1975 to 2005. *J. Clin. Oncol.* 27 1485–1491. 10.1200/JCO.2008.20.7753 19224838 PMC2668555

[B2] Cerami E. Gao J. Dogrusoz U. Gross B. E. Sumer S. O. Aksoy B. A. (2012). The cBio cancer genomics portal: an open platform for exploring multidimensional cancer genomics data. *Cancer Discov.* 2 401–404. 10.1158/2159-8290.CD-12-0095 22588877 PMC3956037

[B3] de Martel C. Maucort-Boulch D. Plummer M. Franceschi S. (2015). World-wide relative contribution of hepatitis B and C viruses in hepatocellular carcinoma. *Hepatology* 62 1190–1200. 10.1002/hep.27969 26146815 PMC5019261

[B4] GBD 2015 Mortality and Causes of Death Collaborators (2016). Global, regional, and national life expectancy, all-cause mortality, and cause-specific mortality for 249 causes of death, 1980-2015: a systematic analysis for the global burden of disease study 2015. *Lancet* 388 1459–1544. 10.1016/S0140-6736(16)31012-1 27733281 PMC5388903

[B5] Giulietti M. Occhipinti G. Principato G. Piva F. (2016). Weighted gene co-expression network analysis reveals key genes involved in pancreatic ductal adenocarcinoma development. *Cell Oncol.* 39 379–388. 10.1007/s13402-016-0283-7 27240826 PMC13001876

[B6] Giulietti M. Righetti A. Principato G. Piva F. (2018). LncRNA co-expression network analysis reveals novel biomarkers for pancreatic cancer. *Carcinogenesis* 39 1016–1025. 10.1093/carcin/bgy069 29796634

[B7] Harris M. A. Clark J. Ireland A. Lomax J. Ashburner M. (2004). The gene ontology (GO) database and informatics resource. *Nucleic Acids Res.* 32 D258–D261. 14681407 10.1093/nar/gkh036 PMC308770

[B8] Hou Y. Wang Z. Huang S. Sun C. Zhao J. (2019). SKA3 Promotes tumor growth by regulating CDK2/P53 phosphorylation in hepatocellular carcinoma. *Cell Death Dis.* 10:929. 10.1038/s41419-019-2163-3 31804459 PMC6895034

[B9] Hu R. Wang M. Q. Niu W. B. Wang Y. J. Liu Y. Y. Liu Y. (2018). SKA3 promotes cell proliferation and migration in cervical cancer by activating the PI3K/Akt signaling pathway. *Cancer Cell Int.* 18:183. 10.1186/s12935-018-0670-4 30459531 PMC6236911

[B10] Huang da W. Sherman B. T. Lempicki R. A. (2009). Systematic and integrative analysis of large gene lists using DAVID bioinformatics resources. *Nat. Protoc.* 4 44–57. 10.1038/nprot.2008.211 19131956

[B11] Ikeda K. Saitoh S. Tsubota A. Arase Y. Chayama K. Kumada H. (1993). Risk factors for tumor recurrence and prognosis after curative resection of hepatocellular carcinoma. *Cancer* 71 19–25. 10.1002/1097-0142(19930101)71:1¡19::aid-cncr2820710105¿3.0.co;2-i 8380116

[B12] Kanehisa M. Sato Y. Kawashima M. Furumichi M. Tanabe M. (2016). KEGG as a reference resource for gene and protein annotation. *Nucleic Acids Res.* 44 D457–D462. 10.1093/nar/gkv1070 26476454 PMC4702792

[B13] Langfelder P. Horvath S. (2008). WGCNA: an R package for weighted correlation network analysis. *BMC Bioinformatics* 9:559. 10.1186/1471-2105-9-559 19114008 PMC2631488

[B14] Li J. Gao J. Z. Du J. L. Huang Z. X. Wei L. X. (2014). Increased CDC20 expression is associated with development and progression of hepatocellular carcinoma. *Int. J. Oncol.* 45 1547–1555. 10.3892/ijo.2014.2559 25069850

[B15] Li K. Wang H. T. He Y. K. Guo T. (2017). New idea for treatment strategies for barcelona clinic liver cancer stages based on a network meta-analysis. *Medicine* 96:e6950. 10.1097/MD.0000000000006950 28514316 PMC5440153

[B16] Li N. Li L. Chen Y. (2018). The identification of core gene expression signature in hepatocellular carcinoma. *Oxid. Med. Cell Longev.* 2018:3478305. 10.1155/2018/3478305 29977454 PMC5994271

[B17] Lin F. Xie Y. J. Zhang X. K. Huang T. J. Xu H. F. Mei Y. (2019). GTSE1 is involved in breast cancer progression in p53 mutation-dependent manner. *J. Exp. Clin. Cancer Res.* 38:152. 10.1186/s13046-019-1157-4 30961661 PMC6454633

[B18] Lister R. Pelizzola M. Dowen R. H. Hawkins R. D. Hon G. Tonti-Filippini J. (2009). Human DNA methylomes at base resolution show widespread epigenomic differences. *Nature* 462 315–322. 10.1038/nature08514 19829295 PMC2857523

[B19] Liu X. Liao W. J. Yuan Q. Ou Y. Huang J. (2015). TTK activates Akt and promotes proliferation and migration of hepatocellular carcinoma cells. *Oncotarget* 6 34309–34320. 10.18632/oncotarget.5295 26418879 PMC4741454

[B20] Marchesi S. Montani F. Deflorian G. D’Antuono R. Cuomo A. Bologna S. (2014). DEPDC1B coordinates de-adhesion events and cell-cycle progression at mitosis. *Dev. Cell* 31 420–433. 10.1016/j.devcel.2014.09.009 25458010 PMC4250264

[B21] Meissner A. Mikkelsen T. S. Gu H. C. Wernig M. Hanna J. Sivachenko A. (2008). Genome-scale DNA methylation maps of pluripotent and differentiated cells. *Nature* 454 766–U791. 10.1038/nature07107 18600261 PMC2896277

[B22] Murakami Y. Yasuda T. Saigo K. Urashima T. Toyoda H. Okanoue T. (2006). Comprehensive analysis of microRNA expression patterns in hepatocellular carcinoma and non-tumorous tissues. *Oncogene* 25 2537–2545. 10.1038/sj.onc.1209283 16331254

[B23] Robinson M. D. McCarthy D. J. Smyth G. K. (2010). edgeR: a bioconductor package for differential expression analysis of digital gene expression data. *Bioinformatics* 26 139–140. 10.1093/bioinformatics/btp616 19910308 PMC2796818

[B24] Su Y. F. Liang C. Y. Huang C. Y. Peng C. Y. Chen C. C. Lin M. C. (2014). A putative novel protein, DEPDC1B, is overexpressed in oral cancer patients, and enhanced anchorage-independent growth in oral cancer cells that is mediated by Rac1 and ERK. *J. Biomed. Sci.* 21:67. 10.1186/s12929-014-0067-1 25091805 PMC4237807

[B25] Tang J. N. Kong D. G. Cui Q. X. Wang K. Zhang D. Gong Y. (2018). Prognostic genes of breast cancer identified by gene co-expression network analysis. *Front. Oncol.* 8:13. 10.3389/fonc.2018.00374 30254986 PMC6141856

[B26] Tang Z. Li C. Kang B. Gao G. Li C. Zhang Z. (2017). GEPIA: a web server for cancer and normal gene expression profiling and interactive analyses. *Nucleic Acids Res.* 45 W98–W102. 10.1093/nar/gkx247 28407145 PMC5570223

[B27] Waghray A. Murali A. R. Menon K. N. (2015). Hepatocellular carcinoma: from diagnosis to treatment. *World J. Hepatol.* 7 1020–1029. 10.4254/wjh.v7.i8.1020 26052391 PMC4450179

[B28] Wallace M. C. Preen D. Jeffrey G. P. Adams L. A. (2015). The evolving epidemiology of hepatocellular carcinoma: a global perspective. *Expert Rev. Gastroenterol. Hepatol.* 9 765–779. 10.1586/17474124.2015.1028363 25827821

[B29] Wang C. Yu L. Ren X. Wu T. Chen X. Huang Y. (2019). The oncogenic potential of PRR11 gene in tongue squamous cell carcinoma cells. *J. Cancer* 10 2541–2551. 10.7150/jca.29265 31258760 PMC6584353

[B30] Wang Y. Chen L. Wang G. Cheng S. Qian K. Liu X. (2019). Fifteen hub genes associated with progression and prognosis of clear cell renal cell carcinoma identified by coexpression analysis. *J. Cell Physiol.* 234 10225–10237. 10.1002/jcp.27692 30417363

[B31] Wilson C. L. Miller C. J. (2005). Simpleaffy: a bioconductor package for affymetrix quality control and data analysis. *Bioinformatics* 21 3683–3685. 10.1093/bioinformatics/bti605 16076888

[B32] Wong N. Yen W. Wong W. L. Wong N. L. Y. Chan K. Y. Mo F. K. (2009). TOP2A overexpression in hepatocellular carcinoma correlates with early age onset, shorter patients survival and chemoresistance. *Int. J. Cancer* 124 644–652. 10.1002/ijc.23968 19003983

[B33] Worns M. A. Galle P. R. (2010). Future perspectives in hepatocellular carcinoma. *Dig. Liver Dis.* 42 (Suppl. 3), S302–S309. 10.1016/S1590-8658(10)60521-X 20547319

[B34] Wu C. X. Wang X. Q. Chok S. H. Man K. Tsang S. H. Y. Chan A. C. Y. (2018). Blocking CDK1/PDK1/beta-Catenin signaling by CDK1 inhibitor RO3306 increased the efficacy of sorafenib treatment by targeting cancer stem cells in a preclinical model of hepatocellular carcinoma. *Theranostics* 8 3737–3750. 10.7150/thno.25487 30083256 PMC6071527

[B35] Zhang Q. Su R. X. Shan C. Gao C. Wu P. (2018). Non-SMC condensin I complex, subunit G (NCAPG) is a novel mitotic gene required for hepatocellular cancer cell proliferation and migration. *Oncol. Res.* 26 269–276. 10.3727/096504017X15075967560980 29046167 PMC7844763

