# Supplementary material for: Identification of 13 Key Genes Correlated With Progression and Prognosis in Hepatocellular Carcinoma by Weighted Gene Co-expression Network Analysis
Source: Front Genet. 2020 Feb 28;11:153. doi: 10.3389/fgene.2020.00153 (PMC7059753; doi:10.3389/fgene.2020.00153)
Supplement: Supplementary file 1 [file Data_Sheet_1.zip › Supporting Legend.docx]

**Supporting Figure Legend:**

Supporting Tab1. The sequences and Tm values of all hub genes primers.

Supporting Tab2. All the hub genes in turquoise and blue module.

Supporting Fig1-5. Overall survival (OS) and disease-free survival (DFS) of 13 hub genes in GEIPA online website.

Supporting Fig6-8. The receiver operating characteristic (ROC) analysis between high histologic grade (III&IV) and low histologic grade (I&II) of 13 hub genes.

Supporting Fig9-10. The methylation information of 13 hub genes in cbioportal online website.

Supporting Fig11-12. The immunohistochemical information between tumor and non-tumor tissues of HCC of 13 hub genes in The Human Protein Atlas public database.

Supporting Fig13-14a. The results of quantitative real-time PCR validation between tumor and non-tumor tissues of HCC of 13 hub genes. P value was calculated by paired t-test. *p<0.05, **p<0.01, ***p<0.001, NS means no significance.

Supporting Fig14b. The results of quantitative real-time PCR validation between high histologic grade (III&IV) and low histologic grade (I&II) in HCC. P value was calculated by t-test. *p<0.05, **p<0.01, ***p<0.001, NS means no significance.
