# Supplementary figures and images for: Identification of 13 Key Genes Correlated With Progression and Prognosis in Hepatocellular Carcinoma by Weighted Gene Co-expression Network Analysis
Source: Front Genet. 2020 Feb 28;11:153. doi: 10.3389/fgene.2020.00153 (PMC7059753; doi:10.3389/fgene.2020.00153)

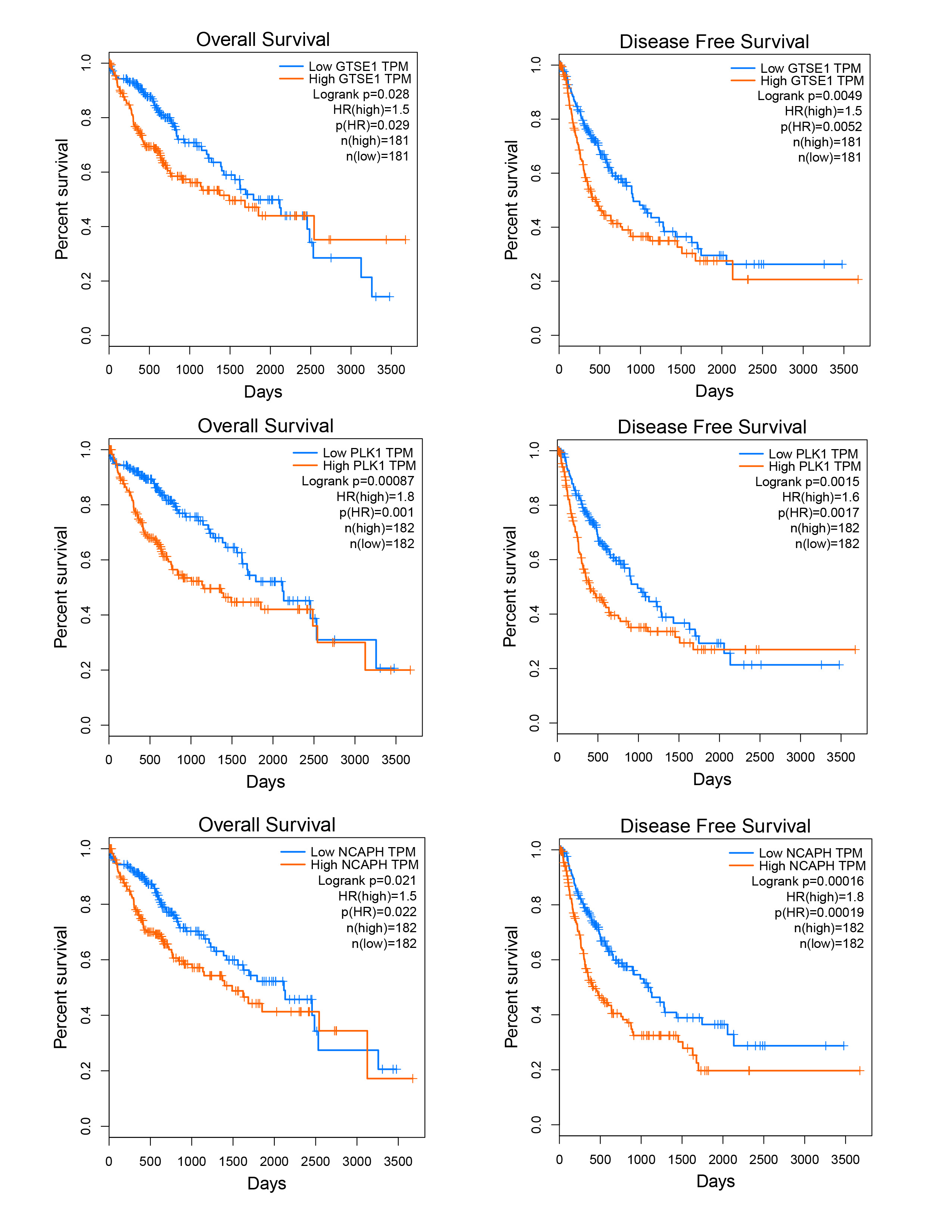

Supplement: Supplementary file 1 [file Data_Sheet_1.zip › Supporting Fig1.jpg]

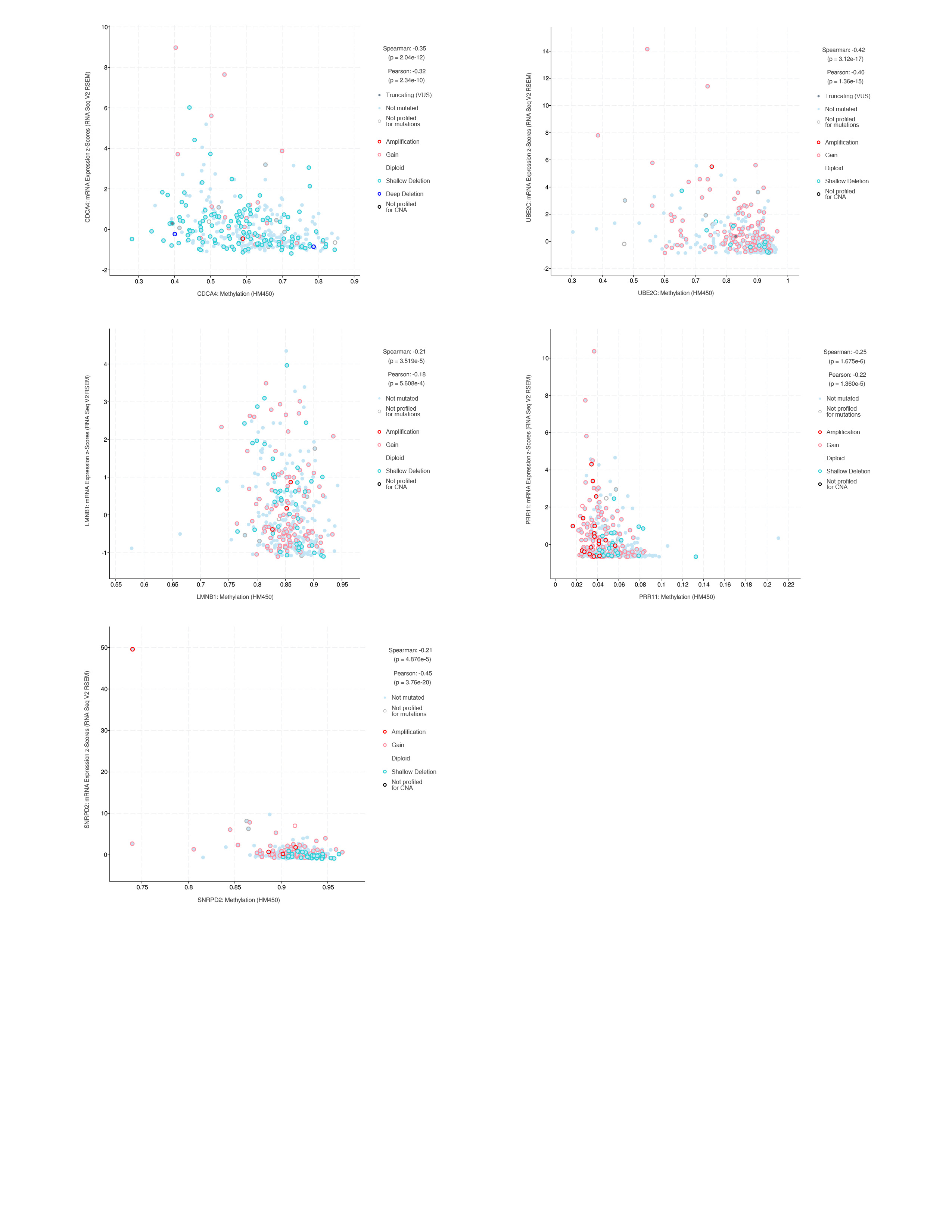

Supplement: Supplementary file 1 [file Data_Sheet_1.zip › Supporting Fig10.jpg]

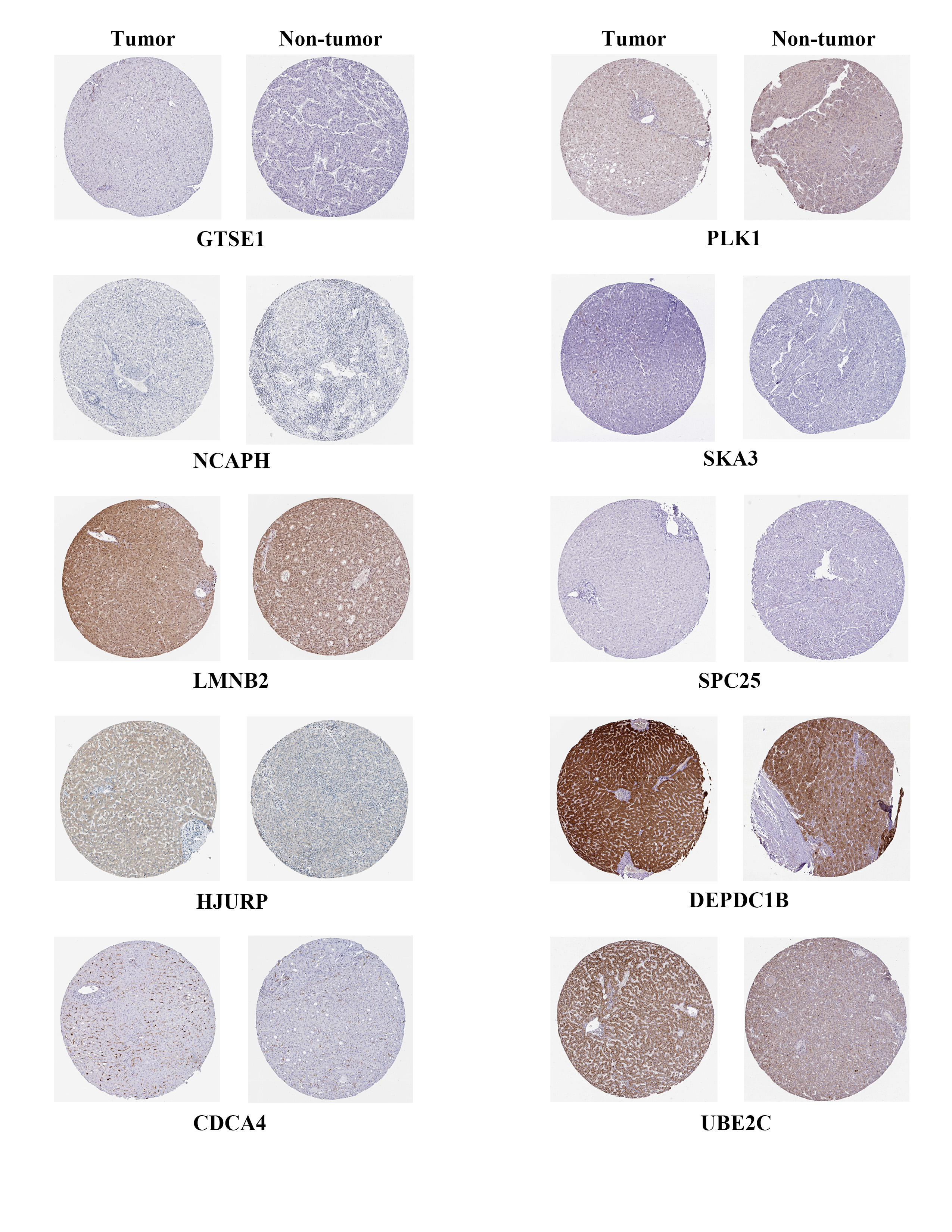

Supplement: Supplementary file 1 [file Data_Sheet_1.zip › Supporting Fig11.jpg]

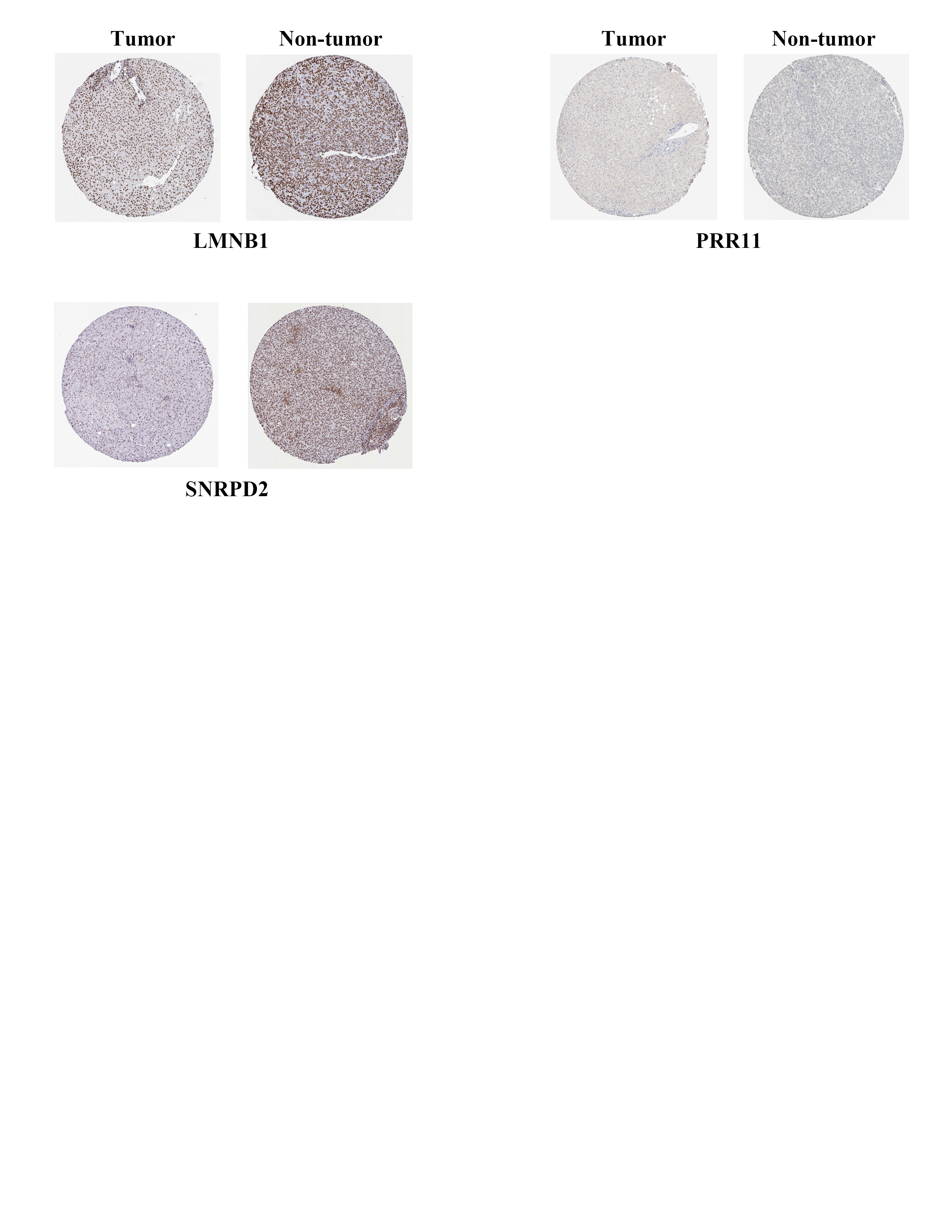

Supplement: Supplementary file 1 [file Data_Sheet_1.zip › Supporting Fig12.jpg]

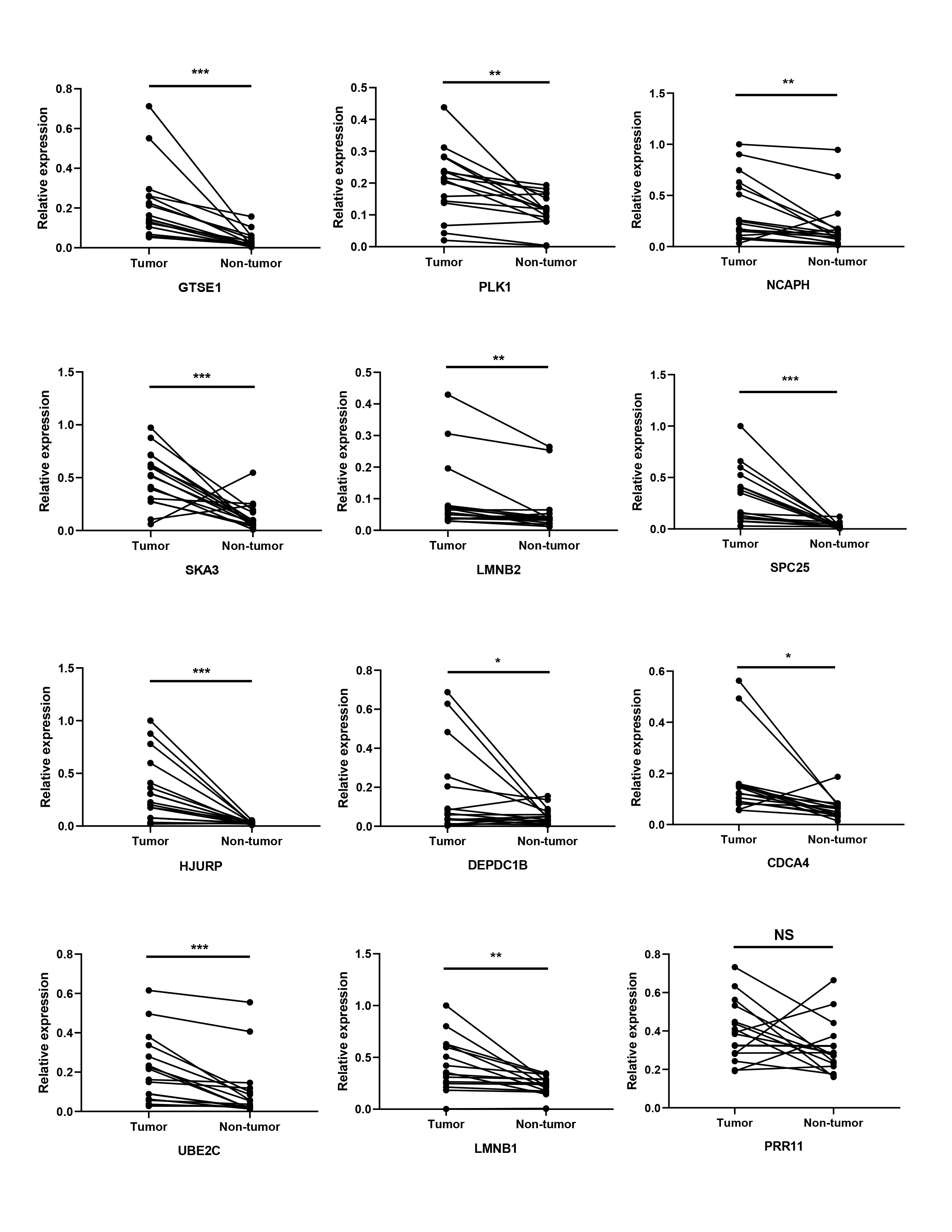

Supplement: Supplementary file 1 [file Data_Sheet_1.zip › Supporting Fig13.jpg]

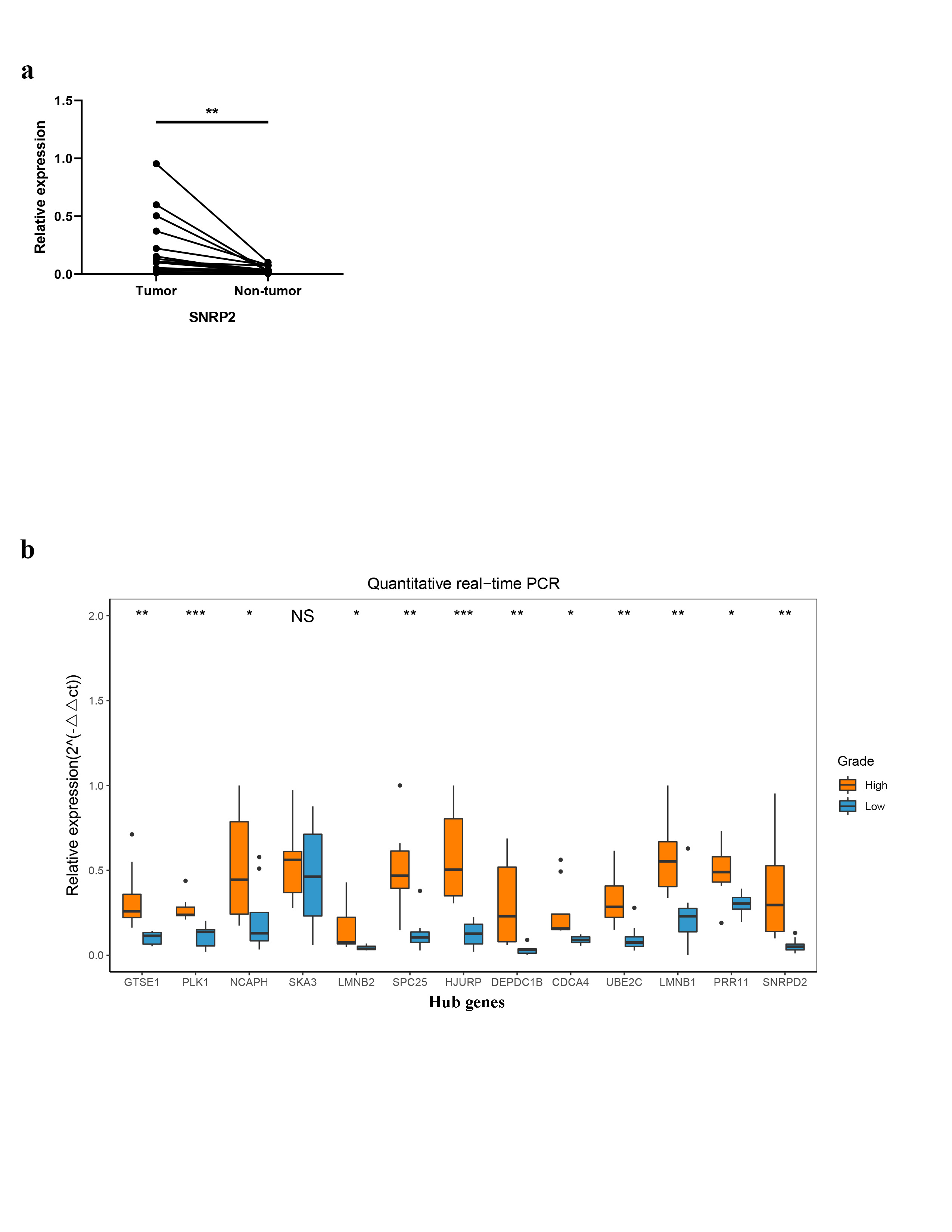

Supplement: Supplementary file 1 [file Data_Sheet_1.zip › Supporting Fig14.jpg]

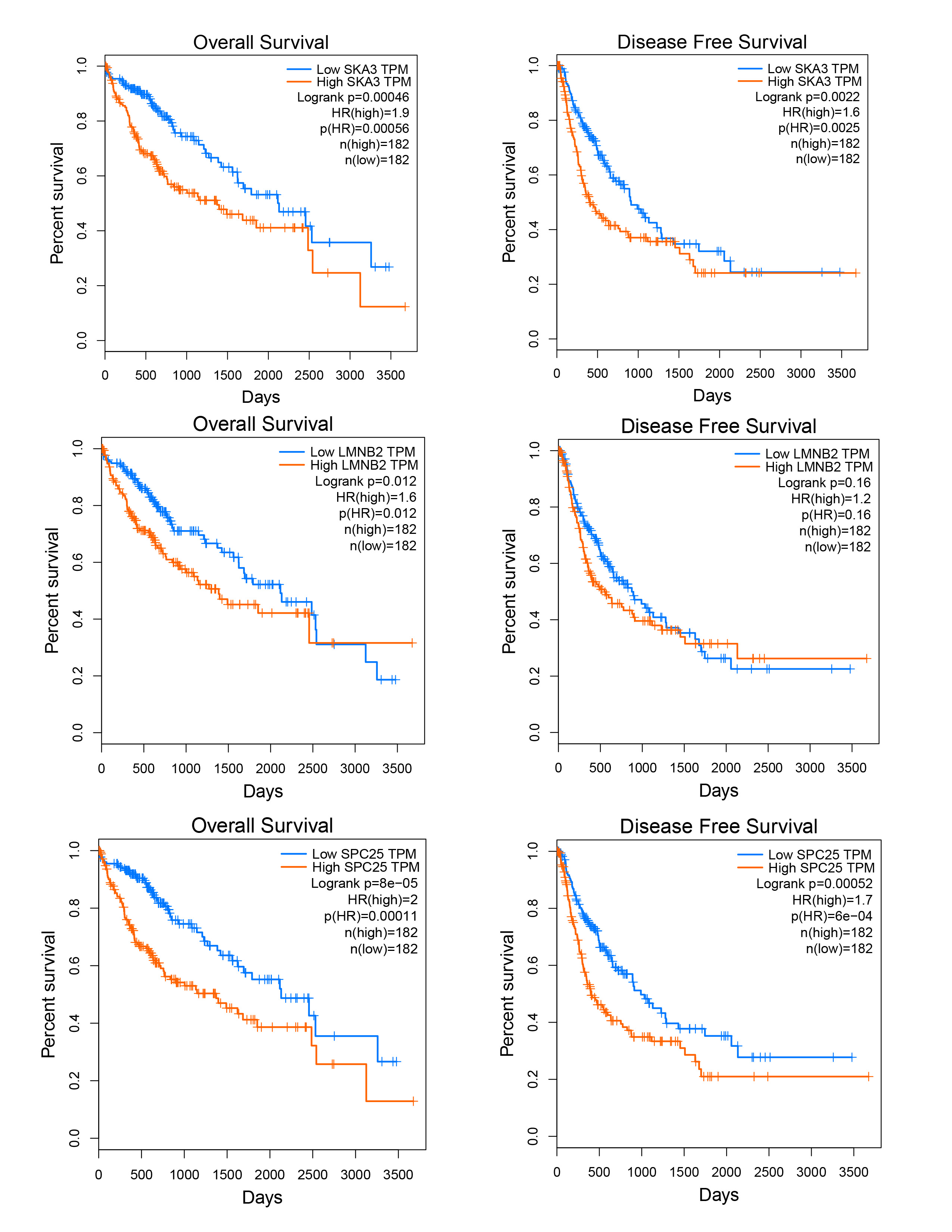

Supplement: Supplementary file 1 [file Data_Sheet_1.zip › Supporting Fig2.jpg]

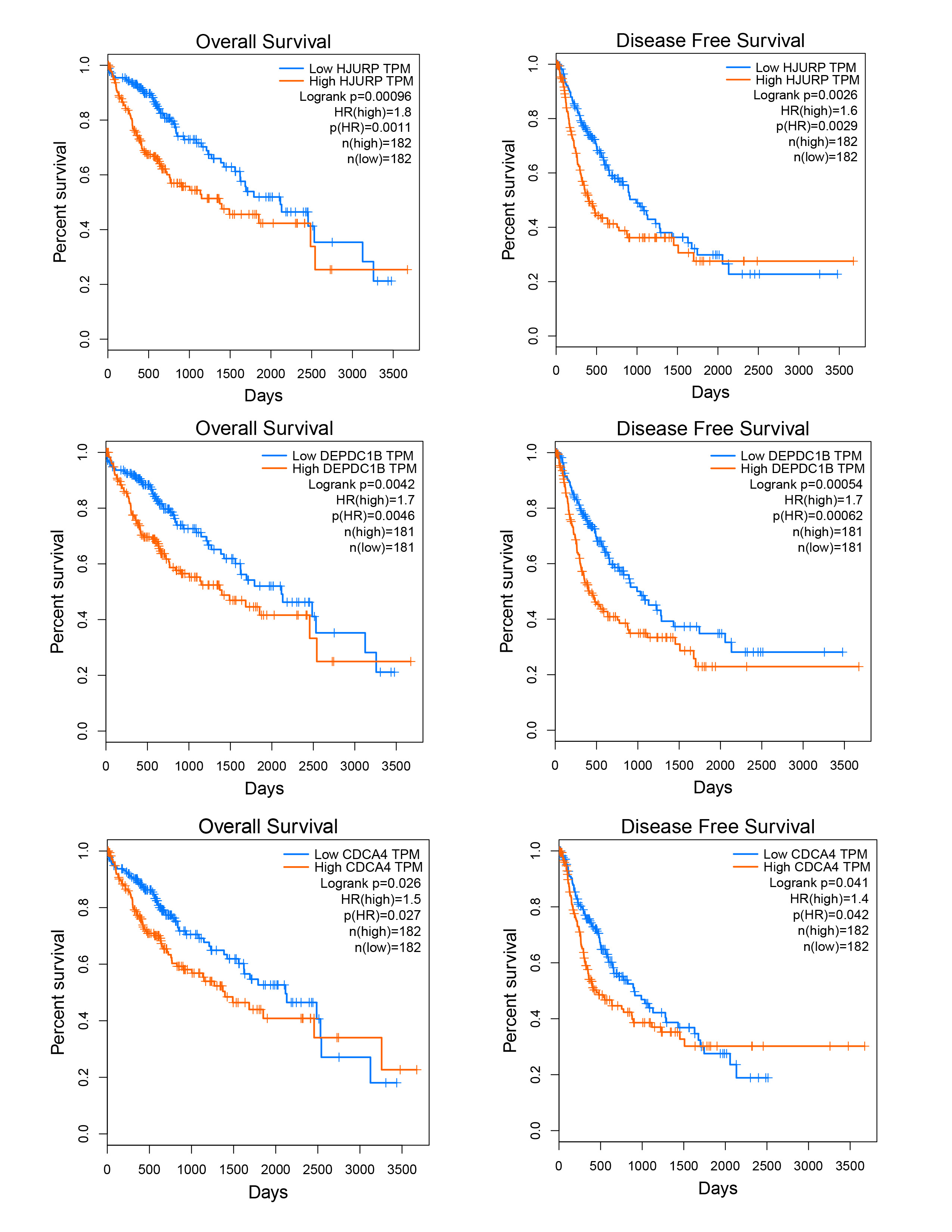

Supplement: Supplementary file 1 [file Data_Sheet_1.zip › Supporting Fig3.jpg]

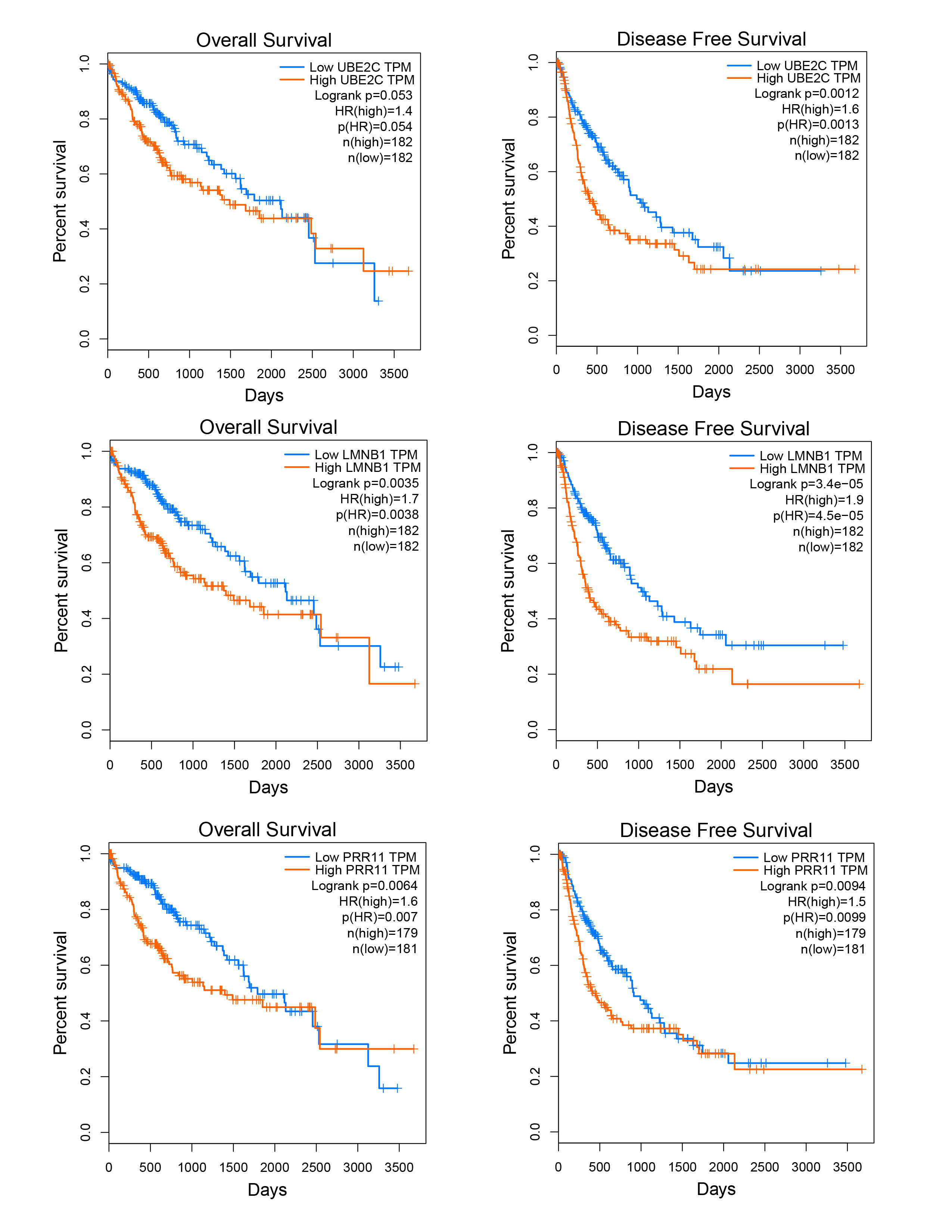

Supplement: Supplementary file 1 [file Data_Sheet_1.zip › Supporting Fig4.jpg]

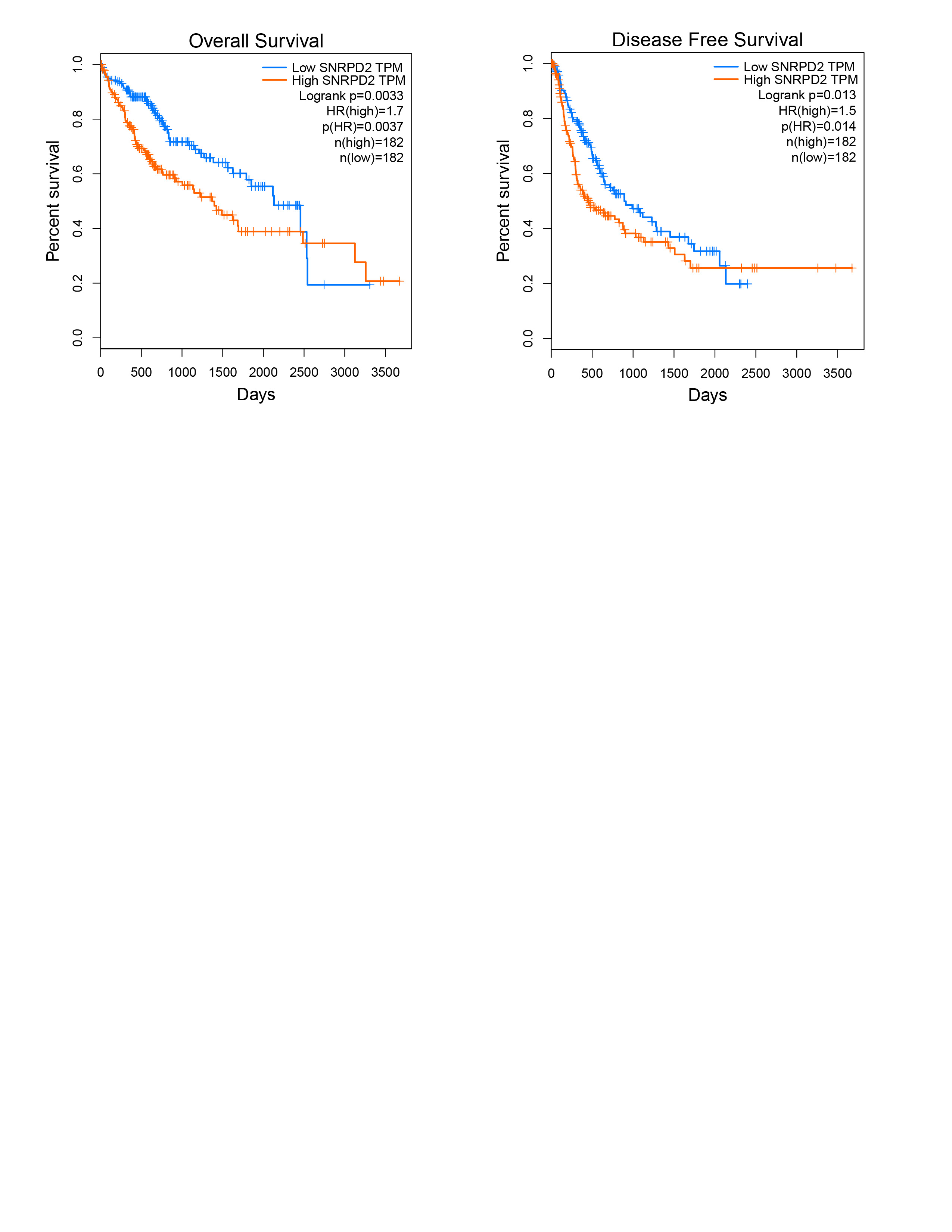

Supplement: Supplementary file 1 [file Data_Sheet_1.zip › Supporting Fig5.jpg]

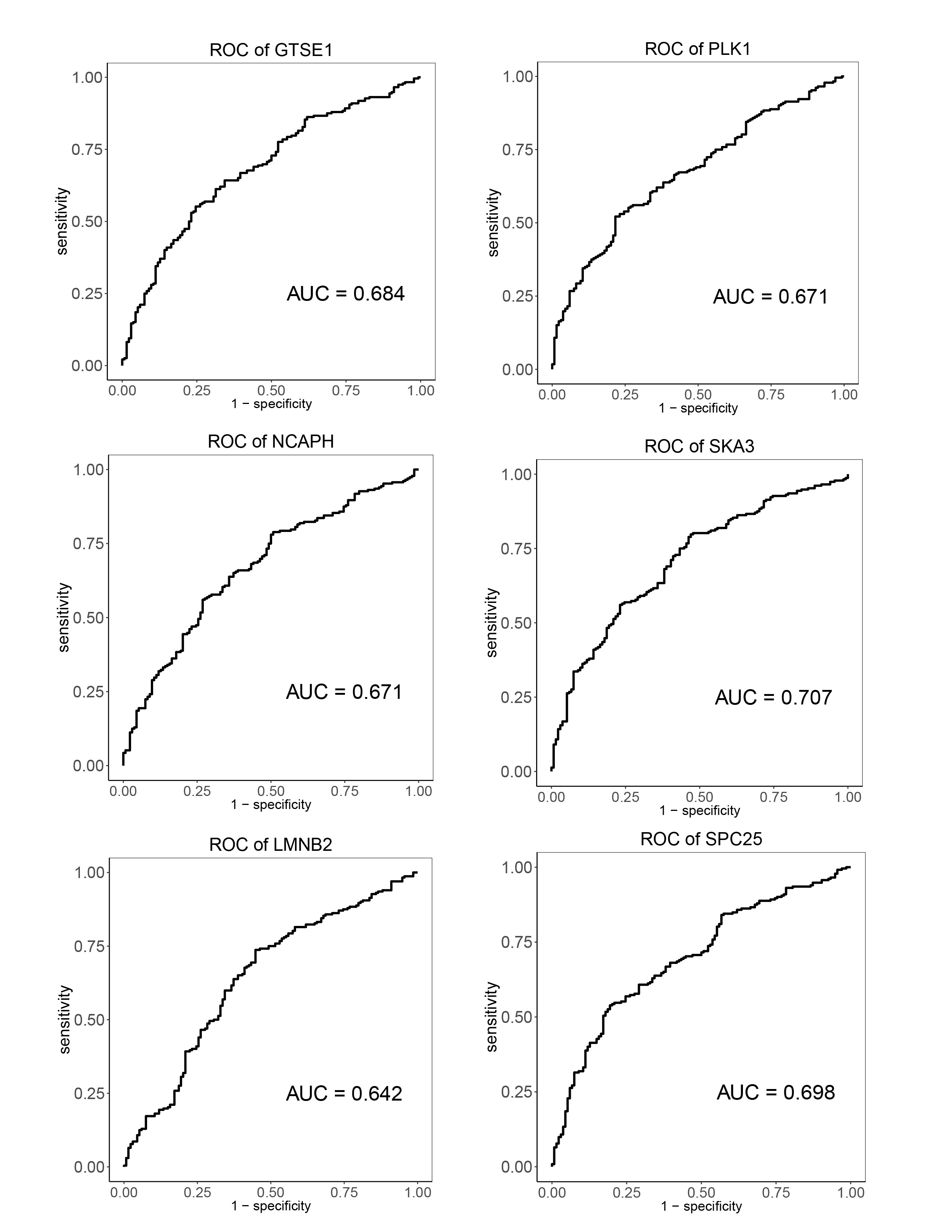

Supplement: Supplementary file 1 [file Data_Sheet_1.zip › Supporting Fig6.jpg]

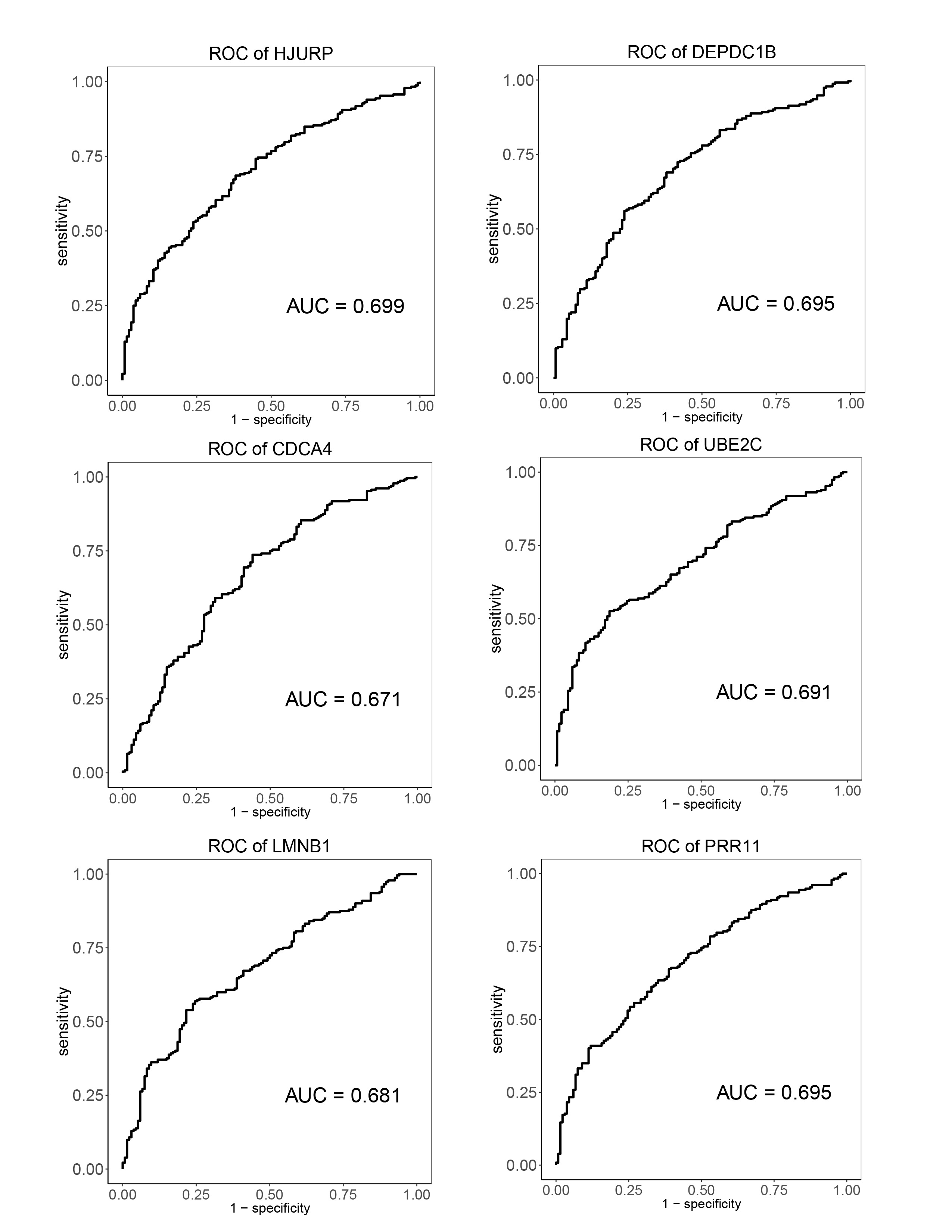

Supplement: Supplementary file 1 [file Data_Sheet_1.zip › Supporting Fig7.jpg]

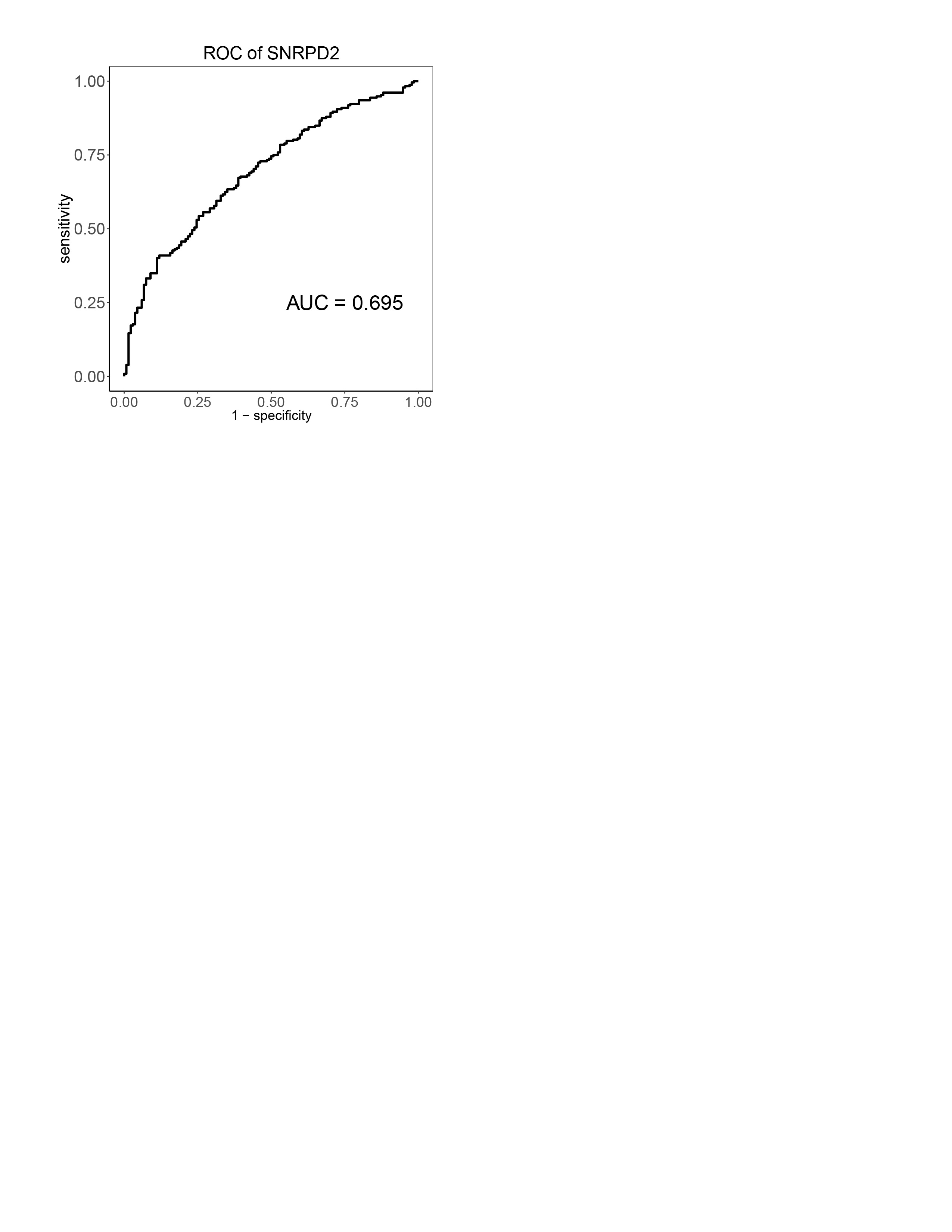

Supplement: Supplementary file 1 [file Data_Sheet_1.zip › Supporting Fig8.jpg]

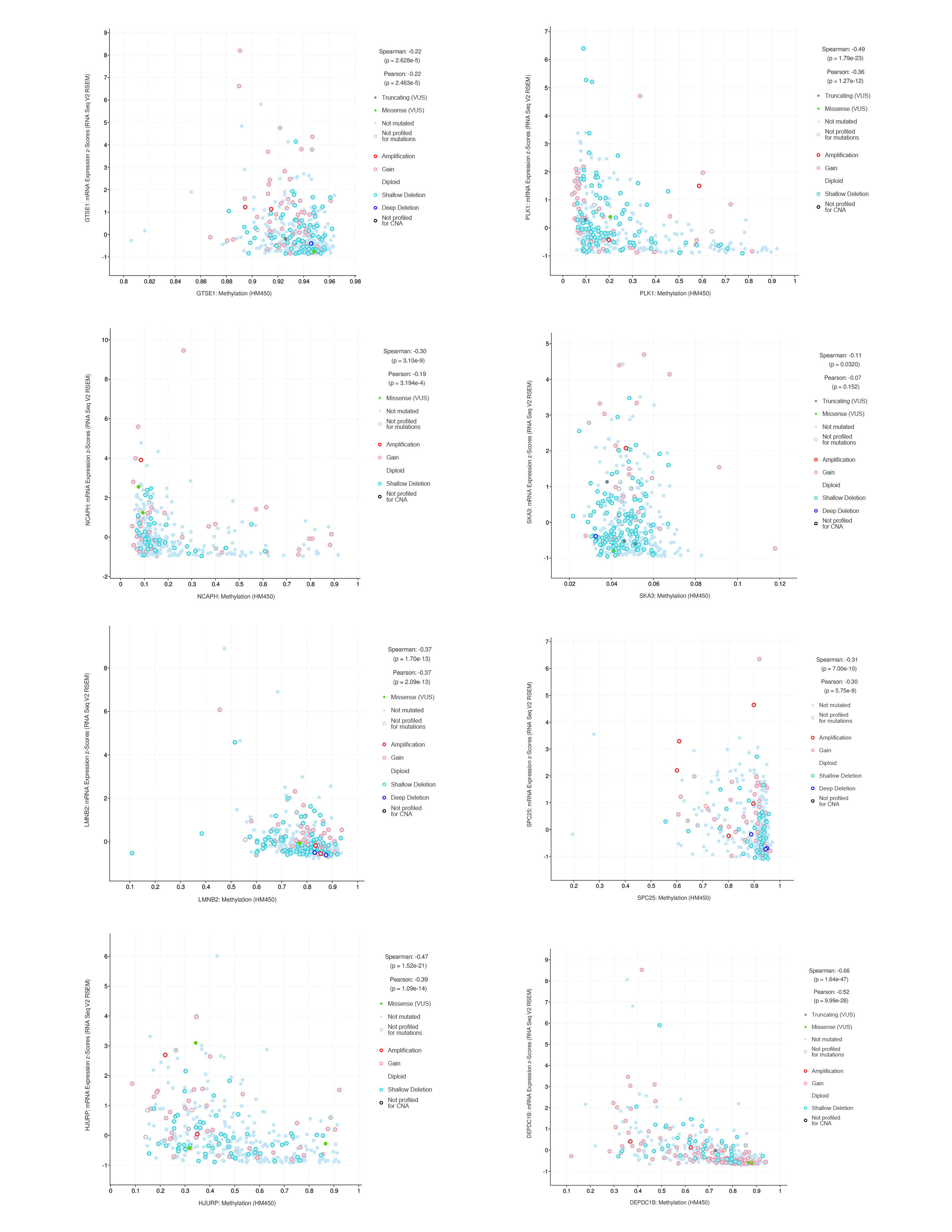

Supplement: Supplementary file 1 [file Data_Sheet_1.zip › Supporting Fig9.jpg]
